# Ultrasensitive Detection of *Staphylococcus aureus* Based on Photonic Crystal Microsphere Suspension Array-Assisted Loop-Mediated Isothermal Amplification

**DOI:** 10.3390/bios16040209

**Published:** 2026-04-09

**Authors:** Xiang Li, Qiaofeng Li, Qianjin Li, Jianlin Li, Zhouping Wang

**Affiliations:** 1 Key Laboratory of Food Science and Resource, Jiangnan University, Lihu Rd 1800, Wuxi 214122, China; lixiang@njucm.edu.cn (X.L.); 7180112016@stu.jiangnan.edu.cn (Q.L.); 2 School of Food Science & Technology, Jiangnan University, Lihu Rd 1800, Wuxi 214122, China; 3 International Joint Laboratory on Food Safety, Jiangnan University, Wuxi 214122, China; 4 School of Health Preservation and Rehabilitation, Nanjing University of Chinese Medicine, Xianlin Rd 138, Nanjing 210023, China; 5 Department of Clinical Laboratory Medicine, Southwest Hospital, Third Military Medical University (Army Medical University), 30 Gaotanyan, Shapingba District, Chongqing 400038, China; 6 School of Food Science and Pharmaceutical Engineering, Nanjing Normal University, Nanjing 210023, China; qianjin_li@njnu.edu.cn

**Keywords:** loop-mediated isothermal amplification, LAMP, photonic crystal microspheres, PCMs, *Staphylococcus aureus*

## Abstract

The development of high-throughput, sensitive and portable strategies for detecting foodborne pathogens is urgently needed in food safety, especially during an outbreak. Herein, an ultrasensitive suspension array was constructed by designing photonic crystal microsphere (PCM)-assisted loop-mediated isothermal amplification (LAMP) for *Staphylococcus aureus* detection. The PCM-LAMP suspension array integrated the optical signal enhancement capability of the biomimetic microporous three-dimensional PCM surface with the thousand-fold signal amplification of LAMP. The biomimetic PCMs displayed a periodic dielectric nanostructure and enhanced the fluorescence intensity of the LAMP reaction, leading to high sensitivity. The PCM-LAMP suspension array allowed sensitive detection of the target DNA of *S. aureus* without long-term culture. Under optimal conditions, the limit of detection for *S. aureus* genomic DNA reached as low as 0.18 fM, and the assay exhibited excellent specificity against other bacteria. Furthermore, trace target DNA in food samples was accurately quantified, demonstrating its potential for practical applications. Therefore, the developed PCM-LAMP suspension array holds great promise for ultrasensitive and rapid detection of foodborne pathogens.

## 1. Introduction

*Staphylococcus aureus* is one of the most common and important pathogenic bacteria and is known as a foodborne pathogen worldwide. Bacterial food poisoning caused by *S. aureus* results in nausea, vomiting and even shock. The traditional quantitative method for *S. aureus* is selective culture and identification. This method has the advantages of simple operation and inexpensive equipment, but it generally has the disadvantages of being time-consuming and having poor sensitivity [1]. Recently, polymerase chain reaction (PCR) and reverse transcriptase-polymerase chain reaction (RT-PCR) have become standard methods for microbial detection. However, they require costly thermal cyclers and multiple steps for the extraction and purification of target genes. To overcome these limitations, isothermal nucleic acid amplification techniques, such as rolling circle amplification (RCA), nucleic acid sequence-based amplification (NASBA), recombinase polymerase amplification (RPA) [2], loop-mediated isothermal amplification (LAMP) [3,4] and helicase-dependent amplification (HDA), were developed. Among these techniques, LAMP is a promising and effective diagnostic tool since it eliminates the need for sophisticated equipment and trained professionals. LAMP has great potential for the detection of DNA, RNA, and proteins in genomics and proteomics. Due to the enzymatic process of LAMP, thousand-fold signal amplification can be achieved. In spite of its many advantages, LAMP still faces formidable challenges in the detection of foodborne pathogens, such as the low efficiency, high cost and insensitivity of single detection, because standard LAMP tests are performed on a single sample, and only one signal can be obtained per assay. In recent years, researchers have been involved in the improvement of these deficiencies. In addition, the limit of detection (LOD) of standard LAMP was around 100 CFU/mL for microorganism detection. Therefore, developing a high-throughput detection method with high sensitivity for detecting foodborne pathogens was urgently needed in food safety assays, especially during a massive outbreak of foodborne pathogens.

A suspension array, also known as a bead-based array or liquid array, is a powerful platform for rapid, sensitive, and high-throughput assays in parallel. Unlike traditional planar microarrays where probes are fixed on a flat surface, a suspension array uses microspheres (or beads) suspended in solution as the solid support for molecular interactions. Typically, the suspension array carrier was solid polystyrene microspheres with a diameter of about 6 µm. Suspension arrays have been used in the fields of nucleic acid, protein and small molecule research. Based on the advantages of suspension microarrays, it is meaningful to design a detection platform based on this technology to establish a detection method for *S. aureus*. However, these methods were mostly used to detect targets based on the interaction between antigen and antibody or to detect the specific signals provided by nucleic acid probes, the sensitivity of which is relatively low while requiring the use of expensive antibodies and fluorescent dyes. The PCR-based suspension array detection platforms have a major advantage over conventional suspension arrays, such as high efficiency. However, multiple steps and dramatic temperature changes are needed for the reactions, as well as complex equipment [5].

Three-dimensional (3D) photonic crystal microspheres (PCMs) are composed of two or more kinds of dielectric materials with different dielectric constants, which self-assemble in an orderly structure and display remarkable enhancement of the intensity of optical species [6,7,8,9]. 3D-PCMs combined with different biological components, such as antibodies, enzymes, gene probes, nucleic acids, and aptamers to identify specific target molecules, can be used in many biosensor fields [10] to enhance signal strength and improve sensitivity hundred-fold [11,12,13,14,15,16,17,18,19]. In the PCM-LAMP biochip system, LAMP, signal enhancement, ultrasensitive and high-throughput detection can be performed in small volumes of reagents. The microarray platforms can provide a powerful tool for rapid detection of foodborne pathogens. Herein, we, for the first time, report a simple, sensitive and rapid suspension array based on LAMP-fluorescent detection using 3D-PCMs for pathogens.

**Scheme 1:**
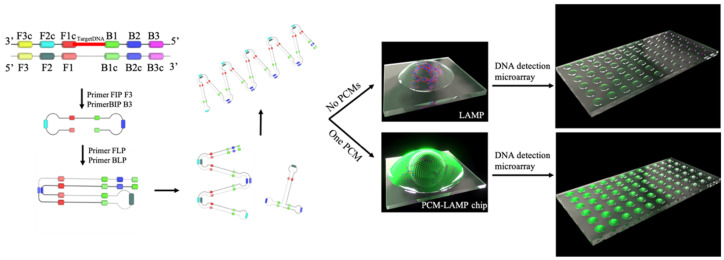
Schematic illustration of the DNA-responsive PCM-LAMP biochip.

Scheme 1 depicts the principle of this process. Three pairs of specific primers rely on the chain replacement of Bst DNA polymerase to allow DNA synthesis to continuously self-cycle and achieve rapid amplification of six regions at the 3′ and 5′ termini of the nucleic acid chain. In this reaction, a dumbbell-shaped template is formed first, and then cyclic amplification is carried out, followed by prolongation and additional cyclic amplification. The nuc gene is a highly conserved DNA sequence of *S. aureus*, which is unique to *S. aureus* [20] and serves as the target gene. If the target contains the nuc gene, the LAMP response can be successfully activated. The nuc gene successfully initiated the LAMP reaction. Due to the excellent signal enhancement capability of the PCMs, in the presence of LAMP amplicons, the fluorescent signal on the surface of PCMs becomes brighter. Based on this strategy, we developed a PCM-assisted LAMP biochip to monitor the presence of *S. aureus* in large-scale real samples.

## 2. Materials and Methods

### 2.1. Materials

Suspensions of silicon nanoparticles with different concentrations were obtained by diluting silicon nanostock with pure water. Dimethylsulfoxide (DMSO, 99%), methyl silicone oil (99%), n-hexane (97%), sodium bicarbonate, and absolute ethanol were purchased from Sinopharm Chemical Reagent Co., Ltd. (Shanghai, China). Absolute ethanol (99.7%), hydrochloric acid (HCl, 36–38%), sodium bicarbonate (99.5%) and ammonium hydroxide (25%, *w*/*w*) were bought from Nanjing Chemicals Ltd. (Nanjing, China). The capillaries (P-727, PEEK TEE0.20 thru hole/F300, and Tub FEP Nat1/16 × 0.20 × 10 ft) and three-way valves were bought from Upchurch Scientific (Oak Harbor, WA, USA). *S. aureus* (CCTCC AB 91093) and *Escherichia coli* O157:H7 (CICC21530) were purchased separately from the China Center for Type Culture Collection (Wuhan, China) and the China Center of Industrial Culture Collection (Beijing, China). *Escherichia coli* and *Pseudomonas* were purchased from the laboratory of Nanjing Institute of Product Quality Inspection. Milk was bought from a local market (Nanjing, China). DNA Hot LAMP MixA3827-03, isothermal amplification buffer and Bst 7.0 WarmStart polymerase were purchased from HaiGene Biotech Co., Ltd. (Harbin, China). SYLGARD^®^ 184 silicone elastomer kit was bought from Sigma-Aldrich (Shanghai, China). Fast pure bacteria DNA isolation mini kit was bought from Vazyme Biotech Co., Ltd. (Nanjing, China). Trypticase soy broth and tryptose soya agar were purchased from Beijing land bridge technology Co., Ltd. (Beijing, China). Petri dishes, coated bars and inoculation loops were bought from Hefei Labgic Technology Co., Ltd. (Hefei, China). Primers were bought from Sangon (Shanghai, China) and listed in the Supplementary Materials (SM). All of the primers were purified by high-performance liquid chromatography (HPLC). A metallographic microscope (MJ33) was used from Guangzhou Mingmei Optoelectronic Technology Co., Ltd. (Guangzhou, China). NanoDrop was used from Thermo Fisher Scientific Inc. (Wilmington, DE, USA).

### 2.2. Bacterial Culture and Genomic DNA Extraction

*S. aureus* was cultured on peptone agar plates. A single colony was transferred from peptone agar plates to peptone broth. A volume of 5 mL of the inoculated broth was incubated at 37 °C for 12 h with constant shaking at 200 rpm. The concentrations of these bacteria were determined by counting cell colonies formed on peptone agar plates. The overnight culture (approximately 10^9^ CFU/mL) was used as the starting solution. It was subjected to serial ten-fold dilutions to achieve a concentration range from 10^9^ to 10^3^ CFU/mL for testing. For the extraction and purification of nucleic acids, a commercially available genomic DNA extraction kit was used. The nuc gene, a well-known target gene for *S. aureus* identification, was selected for detection.

To further investigate the feasibility of detection in real samples, *Staphylococcus aureus* DNA, which was extracted using a commercial genomic DNA extraction kit, was spiked into milk samples.

### 2.3. LAMP Assays

For an off-chip LAMP assay, 20 μL of LAMP reagents were prepared, containing 2 × isothermal amplification buffer (10 μL), 2.5 μL of Bst 7.0 WarmStart polymerase and 2 μL of 10 × LAMP Primer Mix. The nuc gene was added to the LAMP mixture, and the reaction was conducted at 65 °C.

The nuc gene is a highly conserved DNA sequence of *S. aureus*, which encodes the TNase enzyme and is unique to *S. aureus*. The nuc gene sequence of *S. aureus* was obtained from GenBank. Based on the sequence information and previous reports, all the primers were designed using Primer Explorer version 5. Primer sequences are shown in Table S1.

### 2.4. Fabrication of PCMs

The photonic crystal microspheres were fabricated by the droplet-based microfluidic self-assembly method reported by us [21]. The details are provided in Supplementary Materials (Figures S1 and S2).

The PDMS prepolymer was prepared by mixing the base and curing agent of Sylgard 184 silicone elastomer at a 10:1 (*v*/*v*) ratio. After magnetic stirring for 10 min, the mixture was poured onto a glass slide. The mixture was coated on the slide and scraped flat, then dried overnight at 60 °C to fabricate the PDMS-covered substrate. The photonic crystal microspheres can be fixed by placing them vertically on the PDMS substrate.

### 2.5. Simulation of Electric Field Distributions

The electric field distributions of the samples were simulated by commercial software (COMSOL Multiphysics 6.0). The simulated parameters were as follows: the diameters of SiO_2_ were 200, 300, and 350 nm, respectively. A linearly polarized plane wave incident along the *z*-axis was used for excitation at λ = 785 nm.

### 2.6. Detection of LAMP Amplicons

For the detection of *S. aureus*, nuc gene-initiated LAMP amplicons were dropped onto the PCMs that were neatly arranged on the PDMS substrate. The fluorescence images were captured by the microarray chip scanner (LuxScan 10K/D, Beijing, China). Then, the suspension array based on PCMs and the LAMP reaction was successfully established.

### 2.7. Preparation of Standard Curve

A standard curve was constructed to correlate the LAMP signal with bacterial concentration. Briefly, genomic DNA was extracted from a pure culture of *S. aureus* using the fast pure bacteria DNA isolation mini kit. The concentration of the obtained DNA was measured spectrophotometrically (Nanodrop). The bacterial culture used for DNA extraction was serially diluted to determine its precise concentration (CFU/mL). Conversion to theoretical CFU/mL equivalent: The DNA standard was then serially diluted ten-fold. The theoretical equivalent bacterial concentration (CFU/mL) for each DNA dilution point was calculated using the following formula and plotted on a logarithmic scale as the *x*-axis. Theoretical (CFU/mL) = (Initial culture concentration (CFU/mL)) × (DNA dilution factor). This calculation assumes one genome copy is present per colony-forming unit (CFU).

### 2.8. Evaluation of Sensitivity and Specificity

For sensitivity, genomic DNA was diluted in a 10-fold gradient as a template for LOD determination. After the LAMP reaction at 65 °C for 1 h, the LAMP amplicons with PCMs were validated using a microarray chip scanner. For specificity, milk samples spiked with *S. aureus* CCTCC AB 91093, *Escherichia coli* O157:H7, *Escherichia coli*, or *Pseudomonas*, or left blank, were used.

### 2.9. Detection and Recovery Rate of S. aureus in Spiked Food Samples with PCM-LAMP Microarray Biochip

The preparation method of the milk sample is described in the SI. After the sample was prepared, the microbial genomic DNA was extracted from milk using a microbial genome extraction kit. Tap water does not require any treatment, except to ensure that it does not contain *S. aureus.* A volume of 5.5 μL of extracted DNA was added to the LAMP reaction system. The reaction products were further dropped on the PCMs microarray, and the quantification was performed using the above-mentioned procedures. The recovery rate was calculated using the following formula: R (%) = (M − P)/A × 100%, where R is the recovery rate, M is the measured target concentration (e.g., in copies/µL) of the spiked sample, P is the measured target concentration of the original (unspiked) sample, and A is the measured or expected target concentration of the pure standard substance used for spiking.

## 3. Results and Discussion

### 3.1. Suspension Array Detection for S. aureus

Firstly, the droplet-based microfluidic assembly technique was a powerful platform to prepare the 3D-PCMs [22,23,24,25], as shown in the schematic route in Figure S1. The self-assembled photonic crystal microspheres were placed on a PDMS substrate. Figure S2 shows that PCMs self-assembled into a closely packed periodic nanostructure after evaporation. The PCMs have a diameter of 300 µm (Figure S2b) with regular sphericity. Figure S2a shows that SiO_2_ nanoparticles formed the typical face-centered cubic lattice, neatly arranged internally in the SEM image (30 k×). To investigate the feasibility of the LAMP suspension array, LAMP amplicons (10 μM of the nuc gene) were added to the surface of the PDMS substrate with non-PCM and PCM, respectively.

**Figure 1:**
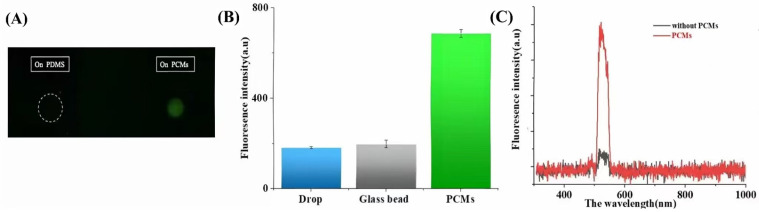
Fluorescence intensity enhancement by photonic crystal microspheres. (**A**) Fluorescence images of LAMP products on the surfaces of bare PDMS and on PCM under excitation with a UV lamp. (**B**) The fluorescence of LAMP products on bare PDMS, glass bead and PCM. (**C**) Comparative fluorescence spectra of the presence and absence of PCM.

The PCMs with the samples on the surface of PDMS were excited by ultraviolet light. Figure 1A shows that the LAMP amplicon on bare PDMS displays weak green fluorescence, whereas bright green fluorescence was clearly observed on the surface of PCMs, indicating that the biomimetic periodic nanostructure of the PCM surface can efficiently enhance the fluorescence intensity of the LAMP amplicon. Furthermore, comparative fluorescence intensities of the PCM and the glass bead with the same diameter are shown in Figure 1B after LAMP amplicons (10 μM of the nuc gene) were added to the surfaces of PCM and the glass bead. The fluorescence intensity of the PCM was much brighter than that of the glass bead, which can be attributed to the efficient reflection of green fluorescence from SYBR Green I by the periodic dielectric nanostructures of the PCM surface (Figure 1B). The fluorescence spectrogram (Figure 1C) of the PCM also shows strong contrast. In Figure 1C, the black curve represents the fluorescence intensity of LAMP amplicons without PCM, while the red curve represents the significantly enhanced fluorescence intensity in the presence of PCM.

### 3.2. Optimization of Photonic Crystal Microspheres

**Figure 2:**
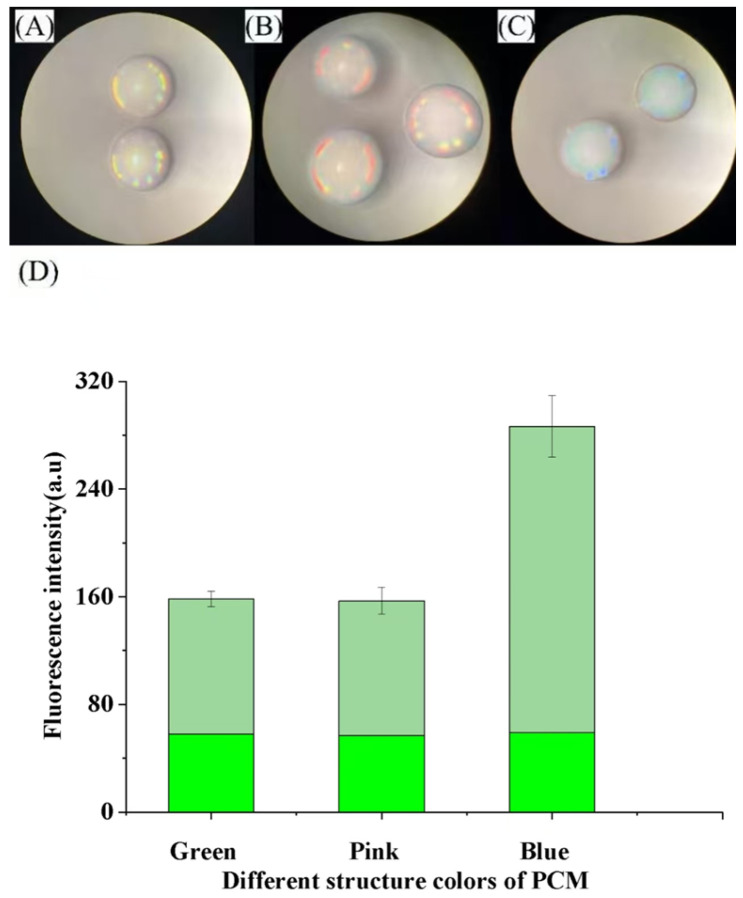
Images from a metallographic microscope showing different structural colors. The PCMs were prepared with different diameters of silica nanoparticles: 300 nm for (**A**) (Green), 350 nm for (**B**) (Pink) and 250 nm for (**C**) (Blue). (**D**) The influence of the different structural colors of PCMs on the fluorescence signal intensity.

The fluorescence intensity signal of the PCM mainly depends on several factors, such as photonic crystal structural color and integration time. We investigated the influence of the structural colors of PCMs on the fluorescence signal enhancement of photonic crystals at a constant integration time. Firstly, we prepared the PCMs with different colors through the self-assembly of SiO_2_ nanoparticles with different diameters. Fluorescence images were acquired using a metallurgical microscope equipped with a UV excitation light source and appropriate filter cubes. The samples were placed on the microscope stage, and UV light was directed onto the sample to excite fluorescence. Emitted fluorescence signals were captured using a CCD camera attached to the microscope. All images were taken under identical settings to ensure consistency. Figure 2A–C shows the bright green, pink, and blue structural colors of PCMs when they were exposed to a metallographic microscope. Figure 2D shows the influence of the different structural colors of PCMs on the fluorescence enhancement effect. When the LAMP amplicons were dropped onto the surfaces of PCMs with different colors, the green and pink structural color PCMs displayed the same fluorescence enhancement effect and the blue PCM showed the maximum fluorescence enhancement effect, which may be attributed to the plasmon resonance on the surface of the PCM.

**Figure 3:**
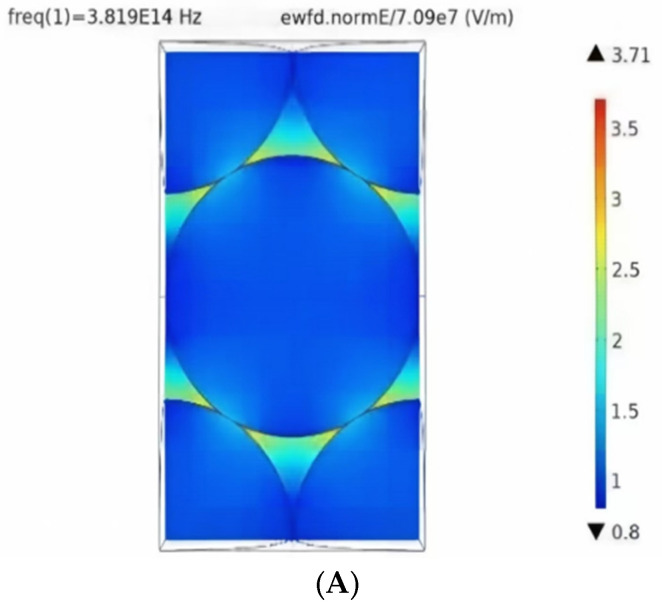

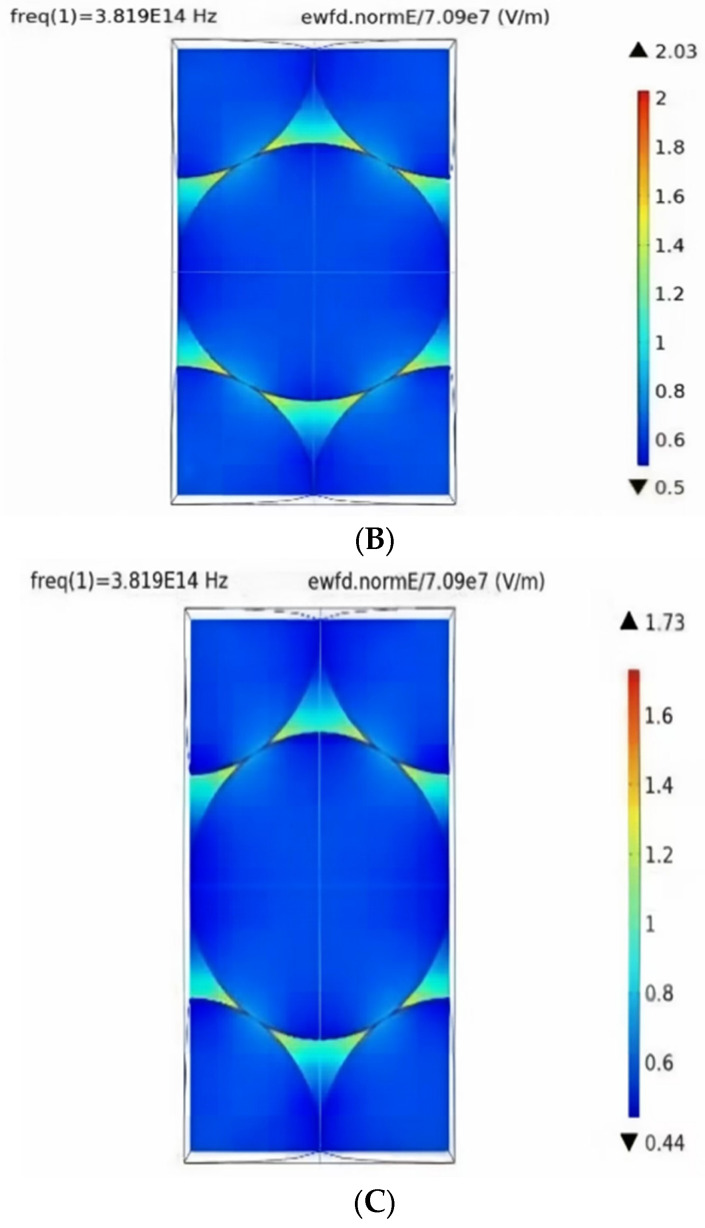
Simulated electric field distributions of the PCM from a vertical view: (**A**) Blue PCM (250 nm SiO_2_ nanoparticles); (**B**) Green PCM (300 nm SiO_2_ nanoparticles); (**C**) Pink PCM (350 nm SiO_2_ nanoparticles).

To investigate the mechanism of the fluorescence enhancement effect of PCMs, the Electric Field (EM) distribution of the PCM was simulated using commercial software (COMSOL Multiphysics) under optimal conditions. The electric field distribution of the 3D photonic crystal microspheres (PCMs) with different structural colors was simulated using the finite element method (FEM) in COMSOL Multiphysics. A 2D axisymmetric model was constructed, consisting of a hexagonal close-packed array of SiO_2_ spheres (refractive index *n* = 1.45) in air (*n* = 1.0). The lattice constants (center-to-center distances) were set to 250 nm, 300 nm, and 350 nm for blue, green, and pink PCMs, respectively, corresponding to their structural colors observed in Figure 3. The electric field enhancement factor (|E|/|E_0_|) was calculated for each structure. As shown in Figure 3, the blue PCMs exhibited the highest near-field enhancement (Δ = 3.71), which was approximately twice that of the green and pink PCMs. This result suggests that the blue PCMs possess a stronger plasmonic resonance effect at the excitation wavelength, leading to greater fluorescence enhancement. Figure 3 shows the maximum value of the near-field enhancements of PCM with different colors. The maximum value of the near-field enhancement reached nearly 4 (Figure 3A) on the blue PCM, which was almost twice that of the green (Figure 3B) and pink (Figure 3C) PCMs. The local electric field intensity distribution of photonic crystals has been confirmed to be positively related to fluorescence intensity [26,27,28]. In detail, the fluorescence enhancement factor (EF) is defined as the ratio of fluorescence intensity observed from the molecule−plasmon system to that from molecules adsorbed on a glass substrate. Generally, photonic crystal-induced photoluminescence enhancement comes from absorption enhancement and emission enhancement [27]. Photonic crystals can increase the utilization of light and enhance the photocurrent of the photodetector. Under the synergistic effects of the photonic crystal effect, the fluorescence intensity has been greatly enhanced [29]. However, the fluorescence effect decreased with increasing silica nanoparticle size of the photonic crystal microspheres. Therefore, microspheres with a structural color of blue were selected for the following experiments.

### 3.3. Optimization of Conditions

**Figure 4:**
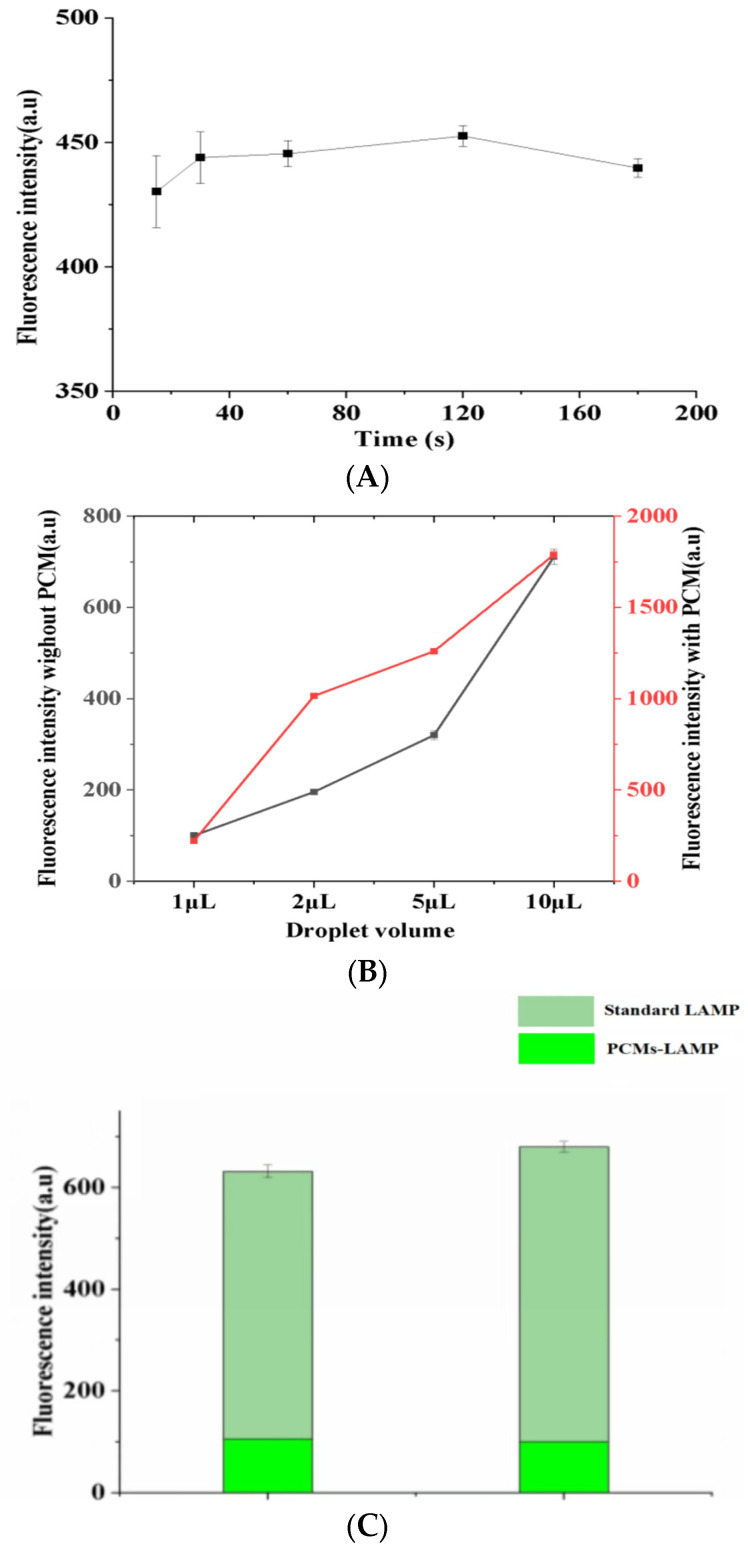
The optimal detection conditions for the developed method. The influence of the different incubation times between the LAMP amplicons and the PCMs on the fluorescence signal (**A**). The influence of different solution volumes of the droplets on the fluorescence signal (**B**). The influence of the different incubation temperatures on the fluorescence signal (**C**).

To establish the optimal conditions for the detection process, the main influencing factors were studied, including the incubation time before detection, the droplet volume, and the reaction temperature. The results are shown in Figure 4. Different detection times ranging from 1 min to 20 min were evaluated to investigate the optimal detection time. When the detection time was greater than 3 min, the droplet dried gradually, which affected the results. We measured the fluorescence intensity at 15, 30, 60, 120 and 180 s. The fluorescence intensity decreased significantly after two minutes, and the light attenuation became more obvious with the extension of time (Figure 4A). Therefore, the optimal incubation time between the LAMP amplicons and the PCM was selected as 2 min. As shown in Figure 4B, the different droplet volumes of 1 μL, 2 μL, 5 μL, and 10 μL were tested for fluorescence enhancement. When the volume of solution was small, the droplets not only dried out during detection but also could not completely suspend the microspheres in the liquid, which resulted in instability and distortion of the fluorescence signal of PCMs and made quantitative detection difficult. The volume of the solution sample was relatively large, which is not helpful for detection in practical operation, and the solution would be prone to flowing and thus affect detection. Therefore, a 2 μL droplet of sample solution was finally selected as the optimal condition. Figure 4C shows the influence of the incubation temperature (4 °C and 25 °C) during the detection on the enhancement of fluorescence intensity, showing that the fluorescence signal at 25 °C incubation was slightly higher than at 4 °C. The results confirmed that photonic crystals significantly outperform microspheres without photonic crystals in enhancing fluorescence intensity. Based on these results, considering that 25 °C is closer to room temperature and easier to operate, the optimal conditions for the new platform are as follows: incubation time before detection of 2 min, sample droplet volume of 2 μL, and reaction temperature of 25 °C. Under optimal conditions, the fluorescence intensities could reach the highest values.

### 3.4. Performance of the DNA-Responsive PCM-LAMP Biochip

The developed DNA-responsive PCM-LAMP biochip contained two steps: the LAMP reaction and PCM signal amplification. The nuc gene successfully initiated the LAMP reaction. Due to the excellent signal enhancement capability of the PCMs, the LAMP reaction on the PCMs surface showed much brighter fluorescence than standard LAMP at the same concentration. Diluted nuc nucleic acid was used as a template for LAMP performed at 65 °C for 1 h. To determine sensitivity, a 10-fold dilution of nuc nucleic acid was examined. The changes in fluorescence intensity caused by different concentrations of *S. aureus* were recorded.

**Figure 5:**
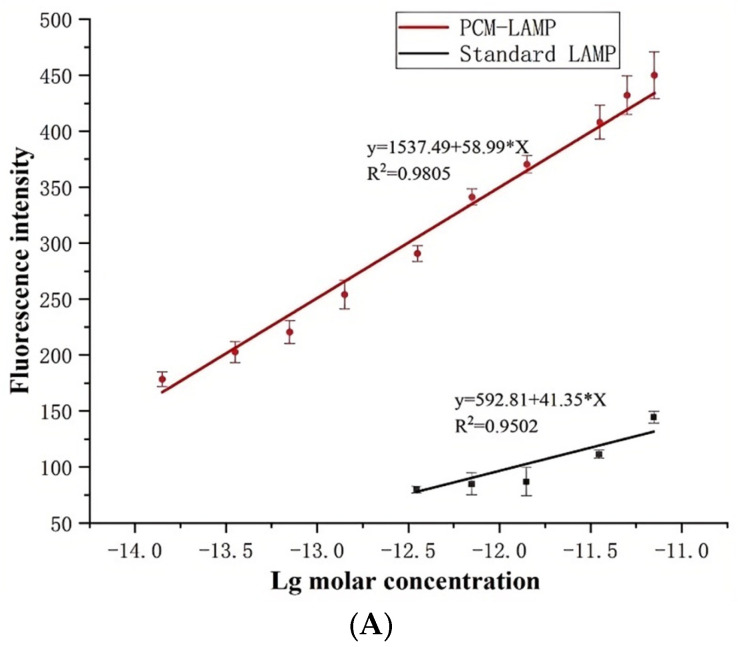

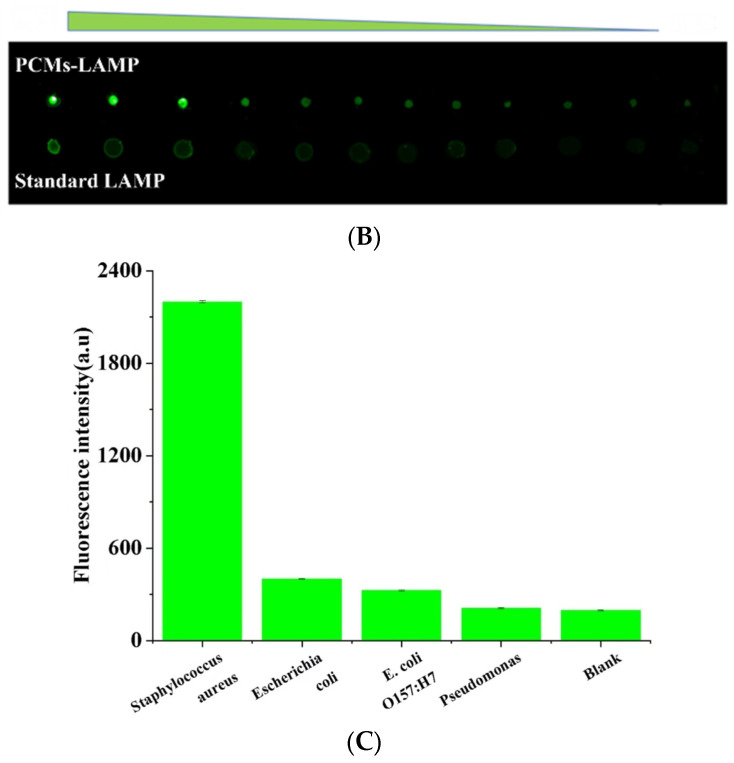
Analytical performance of the LAMP-based detection method for *S. aureus*. (**A**) The relationship between the fluorescence intensity and the concentration of genomic DNA of *S. aureus*. Error bars show the standard deviation of three replicate measurements. (**B**) Fluorescent scanning images of *S. aureus* genomic DNA-initiated LAMP reaction at different concentrations on the PCMs surface (upper panel) and on bare PDMS (lower panel). (**C**) The specificity of the developed method among different microorganisms. Error bars show the standard deviation of three replicate measurements.

As shown in Figure 5A, when the concentrations of nuc ranged from 14.05 fM to 7.02 pM, the fluorescence intensity increased with the increase in the concentration of *S. aureus*. Furthermore, a linear relationship was obtained between the fluorescence intensities and the logarithm of *S. aureus* concentrations from 14.05 fM to 7.02 pM (Figure 5A). The regression equation of the PCM-LAMP liquid-phase biochip in *S. aureus* detection was y= 58.99x + 1537.49, with a coefficient of determination R2 = 0.9805. In Figure 5A, the x-axis shows the logarithm (base 10) of the molar concentration (Lg C). The linear regres-sion line is expressed as y = ax + b, where x = Lg C = log10(C). Therefore, the relationship can also be written as y = a·log10(C) + b. Moreover, a linear range from 14.05 fM (8.46 × 10^6^ CFU/mL) to 7.02 pM (4.23 × 10^9^ CFU/mL) was obtained with the PCM-LAMP liquid-phase biochip in *S. aureus* genomic DNA detection. The results clearly showed that ultrahigh sensitivity could be achieved by combining the signal enhancement function of PCMs and the amplification function of LAMP. The LOD of standard LAMP was calculated as 0.22 μM according to the three signal/noise ratio. The PCM-LAMP liquid-phase biochip showed an LOD of 0.18 fM (1.08 × 10^5^ CFU/mL), indicating ultrahigh sensitivity for *S. aureus* compared with standard LAMP. Figure 5B shows that the fluorescence scanning images of different concentrations of genomic DNA of *S. aureus* on the PCMs and PDMS can be clearly observed by the naked eye, and the fluorescence intensities were quantified by the microarray chip scanner. Obviously, the suspension array on the PCMs greatly enhanced the fluorescence signal. We believe that the developed method could compete effectively with the existing standard LAMP and PCR owing to its ultrahigh sensitivity, simplicity and high-throughput in parallel screening.

The specificity of the PCM-LAMP suspension array was further investigated. The fluorescence responses of the PCM-LAMP suspension array in the presence of different microorganisms were examined. As shown in Figure 5C, the fluorescence intensity of LAMP amplicons on PCMs corresponding to *S. aureus* was nine times higher than that of other microorganisms, suggesting that the PCM-LAMP suspension array could effectively identify *S. aureus*. The results showed that the developed method has good specificity and potential for application in the detection of *S. aureus* in complex samples.

### 3.5. Detection of S. aureus in Real Samples

The application feasibility of the PCM-LAMP suspension array in the detection of *S. aureus* was investigated using spiked milk powder and tap water samples. The target DNA was added to the real samples without *S. aureus* contamination, and the spiking experiment was carried out. Genomic DNA of *S. aureus* at 5.00 × 10^3^ fM, 2.00 × 10^3^ fM and 50.00 fM was spiked into the milk powder and tap water samples, respectively. The incubation time before detection was 2 min. As shown in Table 1, the recovery ratios of the actual samples were in the range of 98.50–102.60%, and the relative standard deviation (RSD) was in the range of 1.70–2.78%, indicating that the new method has good recovery ratios.

## 4. Conclusions

We have developed an ultrasensitive suspension array by designing a PCM-assisted LAMP reaction to achieve signal amplification and high-throughput detection of *S. aureus* genomic DNA. The PCM-LAMP suspension array allowed sensitive detection of the target DNA of *S. aureus* without culturing, and the LOD was as low as 0.18 fM. Moreover, the PCM suspension array possessed excellent specificity. Due to its ultrahigh sensitivity and excellent specificity, trace target DNA in food was accurately quantified by the PCM-LAMP array. The developed PCM-LAMP array has great potential in the ultrasensitive detection of microorganisms, and it can provide a powerful tool for the rapid detection of foodborne pathogens. Admittedly, the current use of a DNA model has its limitations. It is imperative and significant to validate the method using live bacteria in complex food matrices in future studies.

## Figures and Tables

**Table 1:** Detection of *S. aureus* in real samples (*n* = 3).

Samples	Added (fM)	Detected (fM)	Recovery (%)	RSD (%)
Milk powder 1	5.00 × 10^3^	5.13 × 10^3^	102.60	2.78
Milk powder 2	2.00 × 10^3^	2.01 × 10^3^	100.50	2.05
Tap water 1	5.00 × 10^3^	4.98 × 10^3^	99.60	1.75
Tap water 2	2.00 × 10^3^	1.97 × 10^3^	98.50	1.70
Tap water 3	50.00	48.88	97.76	1.91
Tap water 4	50.00	48.82	97.64	1.78

## Data Availability

The data presented in this study are available on request from the corresponding author.

## Supplementary Materials

The following supporting information can be downloaded at: https://www.mdpi.com/article/10.3390/bios16040209/s1. Figure S1. Schematic route for the preparation of PCMs. Figure S2. SEM images of PCMs with opal structure: (a) 30 K×, (b) 1.19 K×. Table S1. Sequences of *S. aureus* LAMP primers. Figure S3. Agarose gel electrophoresis of the LAMP system. Figure S4. Photonic crystal microsphere suspension microarray platform. Table S2. Comparison of rapid LAMP assays for the detection of nucleic acid [30,31,32,33,34,35,36,37,38,39].
