# Supplementary material for: Ultrasensitive Detection of Staphylococcus aureus Based on Photonic Crystal Microsphere Suspension Array-Assisted Loop-Mediated Isothermal Amplification
Source: Biosensors (Basel). 2026 Apr 9;16(4):209. doi: 10.3390/bios16040209 (PMC13113722; doi:10.3390/bios16040209)
Supplement: Supplementary file 1 [file biosensors-16-00209-s001.zip › biosensors-4208804-supplementary.pdf]

Supporting information

Ultrasensitive Detection of *Staphylococcus aureus* Based on Photonic Crystal Microsphere Suspension Array-assisted Loop-mediated Isothermal Amplification

Xiang Li[abce]·Qiaofeng Li[abcf] ·Qianjin Li[d]·Jianlin Li[*d]·Zhouping Wang[*abc]

a Key Laboratory of Food Science and Resource, Jiangnan University, Lihu Rd 1800, Wuxi 214122, Jiangsu, China

b School of Food Science and Technology, Jiangnan University, Lihu Rd 1800, Wuxi 214122, Jiangsu, China

c International Joint Laboratory On Food Safety, Jiangnan University, Wuxi 214122. China.

d School of Food Science and Pharmaceutical Engineering, Nanjing Normal University, Nanjing 210046

e Nanjing University of Chinese Medicine, Xianlin Rd 138, Nanjing 210023, Jiangsu, China

f Department of Clinical Laboratory Medicine, Southwest Hospital, Third Military Medical University (Army Medical University), 30 Gaotanyan, Shapingba District, Chongqing 400038,

*Corresponding authors

Tel.: +86 510 85326195

E-mail addresses:wangzp@jiangnan.edu.cn

SEM images of PCMs self-assembled into a closely packed periodic nanostructure after evaporation.....................3

Sequences of *S. aureus* LAMP primers 3

Recovery ratios for actual sample treatment...4

Agarose gel electrophoresis of the LAMP system...4

Photonic crystal microsphere suspension microarray platform 5

Comparison of rapid LAMP assays for the detection of nucleic acids. 5

Schematic route and method for the preparation of PCMs

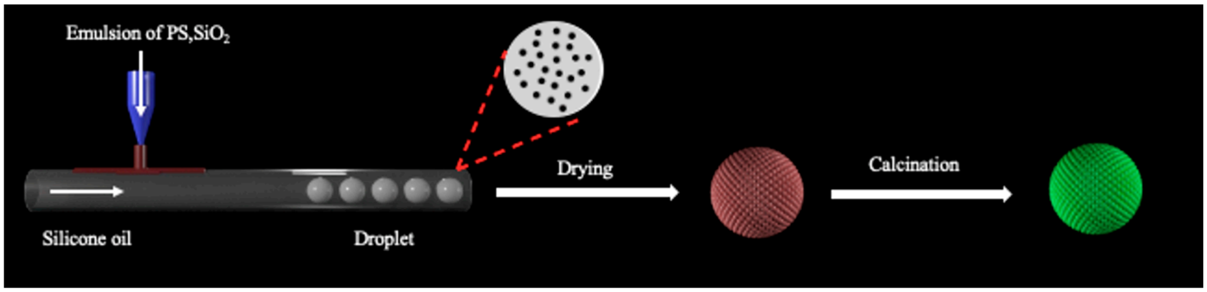

Figure S1. Schematic route for the preparation of PCMs.

The PCMs were fabricated by a droplet-based microfluidic self-assembly system based on the mono-dispersed silica and polystyrene nano-emulsion through a mono-emulsion capillary, which were dispersed in silicon oil. The droplet-based microfluidic assembly system was composed of two pumps, two microfluidic capillary tubes, two syringes, one droplet generator, and a collection container. An aqueous mixture of silica nanoparticles (12%wt) was prepared in double-distilled water and sonicated for 2 h. The flow rate of the dispersed phase was 10 mL/min, and that of the continuous phase was 10 mL/min. The droplets were collected in the silicone oil and heated at 60 °C for over 16 h to form solid microspheres by evaporating water in the droplets. The solid microspheres were washed with n-hexane and ethanol three times, respectively. Finally, the solid microspheres were calcinated at 700 °C for 3 h in a tube furnace with the increasing and decreasing temperature rates sett at 1 °C per minute (Fig. S1).

SEM images of PCMs self-assembled into a closely packed periodic nanostructure after evaporation

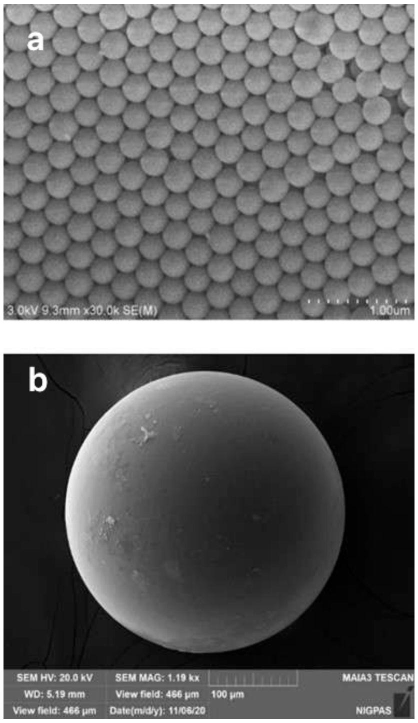

Figure S2. SEM images of PCMs with opal structure: (a) 30 K×; (b) 1.19K×.

Sequences of *S. aureus* LAMP primers

Table S1. Sequences of *S. aureus* LAMP primers

Primer	Sequence (5' to 3')
Nuc-F3	TCG CTT GCT ATG ATT GTG G
Nuc-B3	ACA TAC GCC AAT GTT CTA CC
Nuc-FIP	GTA CAG TTT CAT GAT TCG TCC CGC CAT CAT TAT TGT AGG TGT
Nuc-BIP	TGT TCA AAG AGT TGT GGA TGG TGT ACA GGC GTA TTC GGT T
Nuc-FLP	TTG AAA GGA CCC GTA TGA TTC A
Nuc-BLP	GAT ACG CCA GAA ACG GTG A

Recovery ratios for actual sample treatment.

The milk powder was tested using the plate counting method and used after confirming that it did not contain S. aureus. A total of 25 g milk powder was dissolved in 225 mL of sterilized PBS and thoroughly mixed to prepare a milk powder diluent. S. aureus genomic DNA at different concentrations was employed as the standard solutions. Different concentrations of S. aureus DNA solution were added to the processed milk powder diluent to prepare the artificially contaminated milk powder samples. Protein particles and fat were removed from milk using the rapid microorganism purification kit, and S. aureus was purified. The purified sample solution was obtained, and the response of the PCM-LAMP liquid-phase biochip to the standard solutions was systematically measured.

Agarose gel electrophoresis of the LAMP system

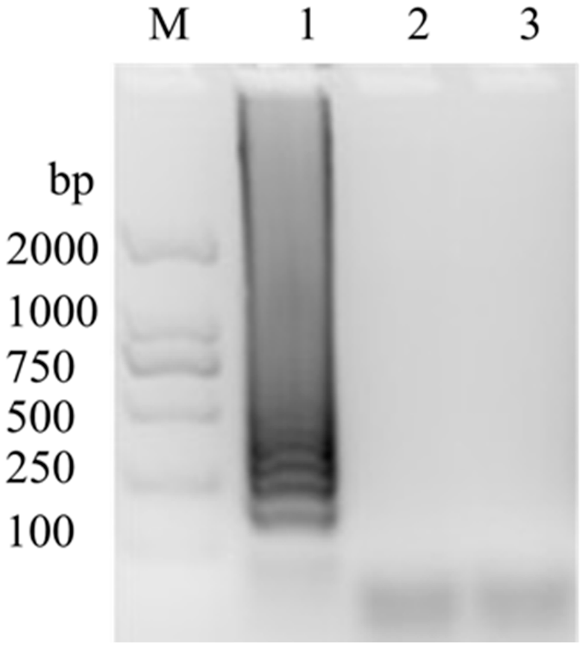

Figure S3. Agarose gel electrophoresis of the LAMP system

Agarose gel electrophoresis of the LAMP system: Lane 1 represents the positive template S. aureus, Lane 2 represents the negative template S. aureus, and Lane 3 represents water without any DNA template as a blank control.

Photonic crystal microsphere suspension microarray platform

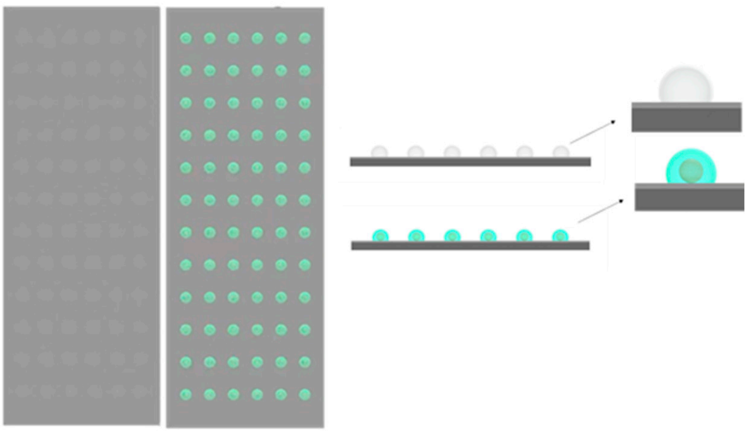

Figure S4. Photonic crystal microsphere suspension microarray platform

Photonic crystal microsphere suspension microarray platform.

Comparison of rapid LAMP assays for the detection of nucleic acids.

Table S2. Comparison of rapid LAMP assays for the detection of nucleic acids.

Indicator	Readout method	Necessary equipment/chemical	Target	On-site	Ref.
Calcein dye	Naked eye, digital colorimetric method by smartphone	Centrifugal LAMP microdevice and smartphone	Escherichia coli, Salmonella, Vibrio	No	[1]
Fluorescent dye	RTFQ	Genie®II, buy LAMP master mix	Plant pathogens	No	[2]
AuNPs or styryl dye	AGE, naked eye, PCR, culture method	AuNPs	SA	No	[3]
Commercial LAMP kit	AGE, naked eye, RTFQ,	Commercial LAMP kit	S. enterica and SA	No	[4]
Commercial LAMP kit	RTFQ, smartphone, naked eye, high-throughput detection by integrated system	CFX96 Touch Real-time PCR Detection System and commercial LAMP kit	Campylobacter coli	No	[5]
Commercial LAMP kit	AGE, naked eye, PCR, LFD, real-time turbidimeter	Loopamp kits and visual detection reagent	Haemophilus influenzae	No	[6]
Phenol red	AGE, naked eye	Phenol red	α-Thalassemia-1	No	[7]
Fluorescent dye FAM	AGE, RTFQ, PCR	Electrophoresis, fluorescent detector	MRSA	No	[8]

phenol red+cresol red	AGE, naked eye, PCR	Phenol red and cresol red	Duck hepatitis B virus	No	[9]
HNB or SYBR Green I	AGE, naked eye, PCR	HNB or SYBR Green I	Colistin resistance gene	No	[10]
PCMs	PCR, fluorescence spectrogram	PCMs	Specific ARG and SA	Yes	Our work

The conventional method for detecting LAMP products is agarose gel electrophoresis, which allows for the easy identification of specific DNA ladder patterns but requires an additional procedure lasting approximately 1 hour. Various strategies exist for detecting LAMP products, each presenting a distinct trade-off between simplicity and analytical performance. Turbidity monitoring offers an instrument-free readout but lacks sensitivity and real-time analysis capabilities. Fluorescence-based detection using DNA intercalating dyes provides real-time data; however, it necessitates a fluorescence spectrophotometer and can be compromised by background interference. Colorimetric detection with metal ion indicators reduces instrument dependency but requires the pre-reaction addition of metal ion-binding reagents, and its sensitivity is often constrained by the limited dynamic range of color change, particularly problematic for trace analysis. However, despite the low cost and ready availability of phenol red, this dye suffers from a limited color transition range (from red to yellow). This subtle color change makes visual interpretation challenging, leading to inaccurate readouts and poor sensitivity, and precludes its use for quantitative analysis. Therefore, achieving a strong fluorescence enhancement is key to optimizing the method's analytical performance.

References

1. Sayad, A.; Ibrahim, F.; Uddin, S.M.; Cho, J.; Madou, M.; Thong, K.L. A microdevice for rapid, monoplex and colorimetric detection of foodborne pathogens using a centrifugal microfluidic platform. *Biosens. Bioelectron.* **2018**, *100*, 96–104, https://doi.org/10.1016/j.bios.2017.08.060.
2. Aglietti, C.; Luchi, N.; Pepori, A.L.; Bartolini, P.; Pecori, F.; Raio, A.; Capretti, P.; Santini, A. Real-time loop-mediated isothermal amplification: an early-warning tool for quarantine plant pathogen detection. *AMB Express* **2019**, *9*, 1–14, https://doi.org/10.1186/s13568-019-0774-9.
3. Srimongkol, G.; Ditmangklo, B.; Choopara, I.; Thaniyavarn, J.; Dean, D.; Kokpol, S.; Vilaivan, T.; Somboonna, N. Rapid colorimetric loop-mediated isothermal amplification for hypersensitive point-of-care Staphylococcus aureus enterotoxin A gene detection in milk and pork products. *Sci. Rep.* **2020**, *10*, 1–11, https://doi.org/10.1038/s41598-020-64710-0.
4. Lu, C.; Qiu, J.; Sun, M.; Liu, Q.; Sakai, E.; Zhang, G. Simple preparation of carboxymethyl cellulose-based ionic conductive hydrogels for highly sensitive, stable and durable sensors. *Cellulose* **2021**, *28*, 4253–4265, https://doi.org/10.1007/s10570-021-03800-2.
5. Lin, Q.; Jia, K.; Gou, H.; He, H.; Wen, J.; Shen, H.; Chen, K.; Wu, Y.; Lu, B.; Liao, M.; et al. A smartphone-assisted high-throughput integrated color-sensing platform for the rapid detection of Campylobacter coli. *LWT* **2022**, *167*, https://doi.org/10.1016/j.lwt.2022.113790.
6. Q. Cao, S. Liang, F. Lin, J. Cao, L. Wang, H. Li, M. Liu, Y. Wang, L. Zhao, X. Cao, BMC microbiology, 22 (2022) 1-12.
7. Chomean, S.; Pholyiam, K.; Thamwarokun, A.; Kaset, C. Development of Visual Detection of α-Thalassemia-1 (the ––^{SEA} Deletion) Using pH-Sensitive Loop-Mediated Isothermal Amplification. *Hemoglobin* **2018**, *42*, 171–177, https://doi.org/10.1080/03630269.2018.1488723.

8. T. Laohasatian, S. Eardmusic, P. Seritrakul. Development of Loop-mediated Isothermal Amplification (LAMP) for rapid detection of Methicillin-resistance Staphylococcus aureus (MRSA) from dairy cattle. March 2022. (PDF) Development of Loop-mediated Isothermal Amplification (LAMP) for rapid detection of Methicillin-resistance Staphylococcus aureus (MRSA) from dairy cattle
9. Ji, J.; Xu, X.; Wu, Q.; Wang, X.; Li, W.; Yao, L.; Kan, Y.; Yuan, L.; Bi, Y.; Xie, Q. Simple and visible detection of duck hepatitis B virus in ducks and geese using loop-mediated isothermal amplification. *Poult. Sci.* **2020**, *99*, 791–796, https://doi.org/10.1016/j.psj.2019.12.024.
10. Liu, Z.; Guo, C.; Zhang, Y.; Zhao, L.; Hao, Z. Rapid and Sensitive Detection of the Colistin Resistance Gene *mcr-3* by Loop-Mediated Isothermal Amplification and Visual Inspection. *Microb. Drug Resist.* **2021**, *27*, 1328–1335, https://doi.org/10.1089/mdr.2020.0129.
